# Interventions to Prevent Unintentional Injuries Among Adolescents: A Systematic Review and Meta-Analysis

**DOI:** 10.1016/j.jadohealth.2016.07.024

**Published:** 2016-10

**Authors:** Rehana A. Salam, Ahmed Arshad, Jai K. Das, Marium Naveed Khan, Wajeeha Mahmood, Stephen B. Freedman, Zulfiqar A. Bhutta

**Affiliations:** a Division of Women and Child Health, Aga Khan University, Karachi, Pakistan; b Ziauddin University, Karachi, Pakistan; c Section of Pediatric Emergency Medicine, Alberta Children's Hospital and Alberta Children's Hospital Research Institute, Cumming School of Medicine, University of Calgary, Calgary, Canada; d Section of Gastroenterology, Alberta Children's Hospital and Alberta Children's Hospital Research Institute, Cumming School of Medicine, University of Calgary, Calgary, Canada; e Centre for Global Child Heath, The Hospital for Sick Children, Toronto, Canada; f Center of Excellence in Women and Child Health, The Aga Khan University, Karachi, Pakistan

**Keywords:** Accidents, Injuries, Adolescent health, Unintentional injuries, Road traffic accidents, Motor vehicle injuries

## Abstract

Globally, every day, ∼2,300 children and adolescents succumb to unintentional injuries sustained from motor vehicle collisions, drowning, poisoning, falls, burns, and violence. The rate of deaths due to motor vehicle injuries in adolescents is 10.2 per 100,000 adolescents. We systematically reviewed published evidence to identify interventions to prevent unintentional injuries among adolescents aged 11–19 years. We defined unintentional injuries as a subset of injuries for which there was no evidence of predetermined intent, and the definition included motor vehicle injuries, suffocation, drowning, poisoning, burns, falls, and sports and recreation. Thirty-five studies met study eligibility criteria. The included studies focused on interventions to prevent motor vehicle injuries and sports-related injuries. Results suggest that possession of a graduated driver license (GDL) significantly reduced road accidents by 19% (relative risk [RR]: .81; 95% confidence interval [CI]: .75–.88; n = 5). There was no impact of GDL programs on incidence of injuries (RR: .78; 95% CI: .57–1.06; n = 2), helmet use (RR: 1.0; 95% CI: .98–1.02; n = 3), and seat belt use (RR: .99; 95% CI: .97–1.0; n = 3). Sports-related injury prevention interventions led to reductions in the incidence of injuries (RR: .66; 95% CI: .53–.82; n = 15), incidence of injury per hour of exposure (RR: .63; 95% CI: .47–.86; n = 5), and injuries per number of exposures (RR: .79; 95% CI: .70–.88; n = 4). Subgroup analysis according to the type of interventions suggests that training ± education and the use of safety equipment had significant impacts on reducing the incidence of injuries. We did not find any study focusing on interventions to prevent suffocation, drowning, poisoning, burns, and falls in the adolescent age group. The existing evidence is mostly from high-income countries, limiting the generalizability of these findings for low- and middle-income countries. Studies evaluating these interventions need to be replicated in a low- and middle-income country–context to evaluate effectiveness with standardized outcome measures.

Injuries are defined as damage to a person caused by an acute transfer of mechanical, thermal, electrical, chemical, or radiation energy or by the sudden absence of heat or oxygen [1]. Unintentional injuries consist of the subset of injuries for which there is no evidence of predetermined intent and include motor vehicle injuries, suffocation, drowning, poisoning, burns, falls, and sports and recreation [1]. Worldwide, unintentional injuries are the second leading cause of years lost because of disabilities for 10- to 24-year-olds accounting for 12% of the total years lost because of disabilities in this age group [2]. Every day nearly 2,300 children and adolescents die from injuries sustained from motor vehicle injuries, drowning, poisoning, falls, burns, and violence while motor vehicle injuries alone are responsible for 10.2 deaths per 100,000 adolescents [3]. Overall, more than 95% of all injury-related deaths occur in low- and middle-income countries (LMICs) in all age groups. In high-income countries (HICs), injuries account for more than 40% of all deaths among children and adolescents [3]. Many of those who do not die due to these injuries are at an increased risk of lifelong disabling health consequences [4], [5]. Furthermore, the impact of these injuries is not limited to physical consequences but also encompasses psychosocial and financial consequences that extend beyond the injury victim [6].

With progress in preventing infectious diseases, there has been a shift in epidemiological patterns with injuries accounting for 9% of global mortality; injuries are a threat to health worldwide [7]. Data indicate an increase in the global burden of injuries with the clear potential to increase steadily if measures are not taken to prevent unintended injuries [7]. Unfortunately, awareness of the problem, the means to prevent it, and the political commitment to act remain unacceptably low [3]. The first global report that brought attention to the issue of child injury prevention was published in December 2008 by the World Health Organization (WHO) and the United Nations Children's Fund [8]. The evidence base for unintentional injury prevention is limited, especially in LMICs; however, some countries have implemented strategies in the form of legislation, product and environment modifications, safety devices, and education to prevent injuries [8]. These interventions target behavioral changes to prevent unintentional injuries (including increased use of safety equipment, seat belt use, helmet use etc.) along with consequent reduction in unintentional injuries. Existing systematic reviews on unintentional injury prevention involve parent injury prevention education and training programs [9], interventions to prevent sports-related injuries [10], home safety education, the provision of safety equipment for injury prevention [11], bicycle helmet legislation [12], and school-based driver education for the prevention of traffic crashes [13]. Existing reviews have either focused on the effectiveness of certain specific interventions or do not target the adolescent age group (11–19 years).

This article is part of a series of reviews conducted to evaluate the effectiveness of potential interventions for adolescent health and well-being. Detailed framework, methodology, and other potential interventions are discussed elsewhere [14], [15], [16], [17], [18], [19], [20]. Our conceptual framework depicts the individual and general risk factors through the life cycle perspective that can have implications at any stage of life [14]. We acknowledge that interventions directed toward parents also have an impact on preventing unintentional injuries among children and adolescents. However, the focus of our review is to evaluate potential interventions directly targeted toward adolescents only and its impact on quality of life. With this focus, we systematically reviewed the evidence regarding interventions to prevent unintentional injuries among adolescents.

## Methods

We systematically reviewed published literature up to December 2014 to identify studies on interventions to prevent unintentional injuries among adolescents, defined as all individuals between the ages 11 and 19 years. We defined unintentional injuries as a subset of injuries for which there is no evidence of predetermined intent; these included motor vehicle injuries, suffocation, drowning, poisoning, burns, falls, and sports- and recreation-related injuries. Studies that did not specifically report outcomes for adolescents or had overlapping age groups were excluded. Eligible study designs included randomized controlled trials (RCTs), quasirandomized, and before/after studies, in which the intervention was directed toward the adolescent population. We did not restrict our search to publication dates or geographical settings. A separate search strategy was developed for each aspect using appropriate keywords, medical subject heading, and free text terms. Key search words included “adolescents, teenagers, youth, injury, accident, license, training, education, driving, burns, fall, drown* and suffocate/ion.” The following principal sources of electronic reference libraries were searched to access the available data: the Cochrane Library, Medline, PubMed, Popline, LILACS, CINAHL, Embase, World Bank's JOLIS search engine, CAB Abstracts, British Library for Development Studies at IDS, the WHO regional databases, Google, and Google Scholar.

The titles and abstracts of all studies identified were screened independently by two reviewers for relevance and matched. Any disagreements on selection of studies between these two primary abstractors were resolved by the third reviewer. After retrieval of full texts of studies that met the inclusion/exclusion criteria, data from each study were abstracted independently and in duplicate into a standardized form. Quality assessment of the included RCTs was done according to the Cochrane risk of bias assessment tool [21].

A meta-analysis of individual studies was performed. The results of comparisons between the experimental and control groups are reported as relative risks (RRs) for categorical variables and standard mean differences for continuous variables. The analysis included all outcomes as reported by study authors of the eligible articles. The pooled statistics were reported using Mantel–Haenszel (M-H) pooled method or DerSimonian–Laird method where there was an unexplained heterogeneity. Heterogeneity was quantified by χ^2^ and *I*^2^; a low *p* value (less than .1) or a large chi-square statistic relative to its degree of freedom and *I*^2^ values greater than 50% were taken as substantial and high heterogeneity. In situations of high heterogeneity, causes were explored by sensitivity analysis and random effect models were used. All analyses were conducted using Review Manager, version 5.3 (Cochrane Collaboration, London, United Kingdom), which is a freely downloadable software used for conducting meta-analysis and presenting results graphically [22]. For all outcomes, the analysis was conducted employing the intention-to-treat principal. Our primary comparison was to evaluate the effectiveness of any interventions to prevent unintentional injuries among adolescents compared to no intervention or standard care; however, where possible, we attempted to conduct subgroup analysis according to the type of interventions.

The overall evidence indicating the strength of an effect on specific health outcome was assessed employing the Grading of Recommendations Assessment, Development and Evaluation (GRADE) criteria [23] which include the following categories: “high,” “moderate,” “low,” and “very low.” The GRADE Working Group has developed a system for grading the quality of evidence which is currently recommended by over 20 organizations including the WHO, the American College of Physicians, the American College of Chest Physicians, the American Endocrine Society, the American Thoracic Society, the Canadian Agency for Drugs and Technology in Health, BMJ Clinical Evidence, the National Institute for Health and Clinical Excellence in the United Kingdom, and UpToDate in its original format or with minor modifications [21]. The GRADE approach specifically assesses methodological flaws within the component studies, consistency of results across different studies, generalizability of research results to the wider patient base and how effective the treatments have shown to be (Box 1) [21].

## Results

**Figure 1:**
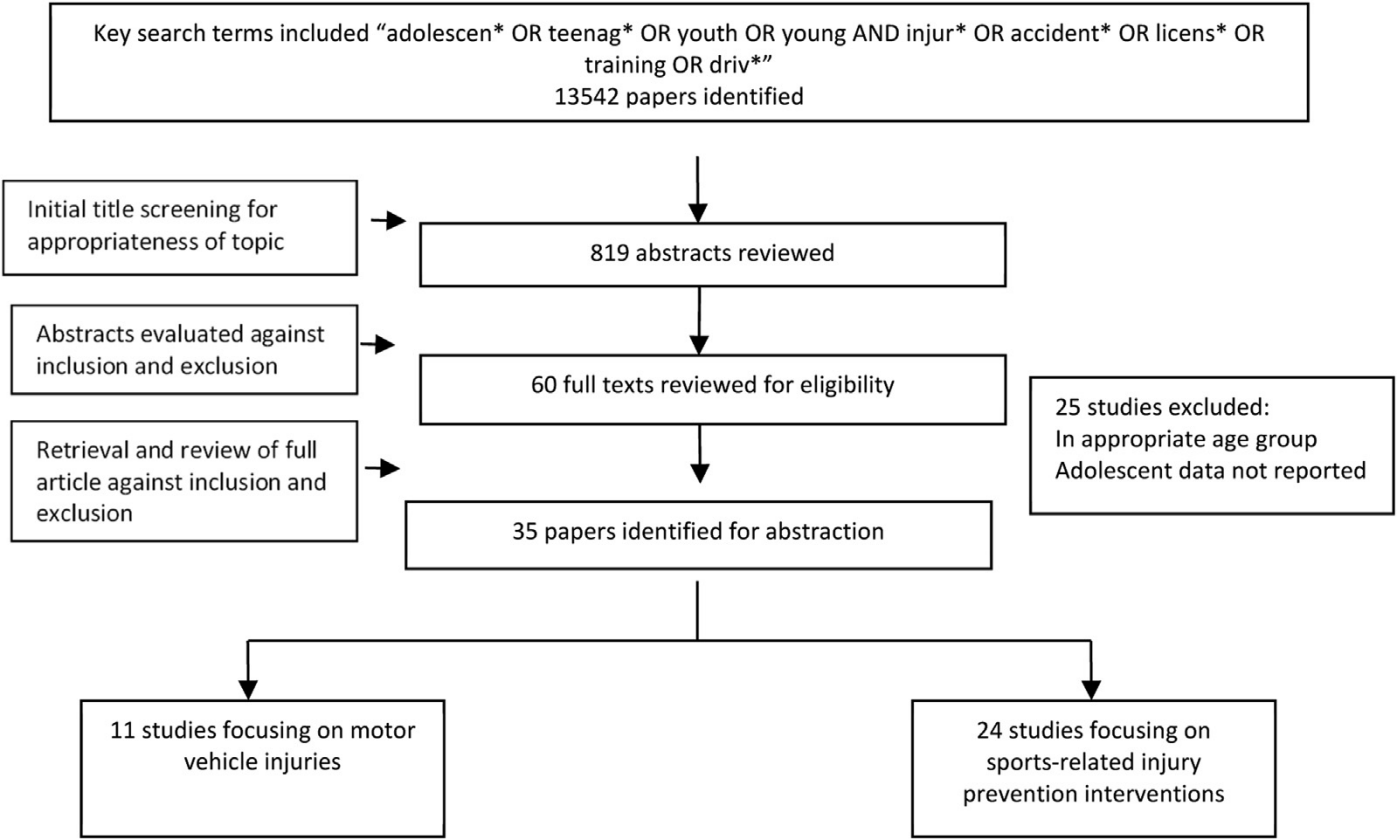
Search flow for interventions to prevent unintentional injuries in adolescents.

The search conducted for this review yielded 13,542 titles that were screened by two independent reviewers. Of these, 60 full texts were retrieved and further screened, and 35 studies were finally included (Figure 1). Of these 35 studies, 7 were before–after studies [24], [25], [26], [27], [28], [29], [30] and 28 were controlled trials [31], [32], [33], [34], [35], [36], [37], [38], [39], [40], [41], [42], [43], [44], [45], [46], [47], [48], [49], [50], [51], [52], [53], [54], [55], [56], [57], [58]. Of the trials included in this review, 19 were adequately randomized [32], [33], [37], [38], [39], [40], [41], [44], [45], [46], [47], [48], [50], [53], [54], [55], [56], [57], [58], and all controlled trials had appropriate control groups. Assessment was blinded in nine of the included trials [38], [39], [44], [45], [50], [52], [53], [55], [57] while selective outcome reporting (outcomes mentioned in the protocol/methods but not in the results section) was identified in two studies. With the exception of Brazil, all included studies were conducted in HICs including USA, Canada, Australia, Switzerland, Sweden, and Norway. Eleven of the included studies were conducted in a local school [24], [27], [28], [30], [32], [33], [34], [37], [38], [39], [41], [44], [46], [51], 19 were conducted in community settings [25], [26], [29], [35], [36], [40], [42], [43], [45], [48], [49], [50], [52], [53], [54], [55], [56], [57], [58], and the remaining studies were conducted in hospital settings [31].

Included studies were classified as those evaluating interventions to prevent motor vehicle injuries or sports-related injuries. We did not find any study focused on interventions to prevent suffocation, drowning, poisoning, burns, and falls among the adolescent age group (ages 11–19 years). A detailed description of the characteristics of included studies can be found in Table 1; Tables 2 and 3 summarize the quality of evidence for motor vehicle injury prevention interventions and sports-related injury prevention, respectively.

### Interventions for motor vehicle injury prevention

**Figure 2:**
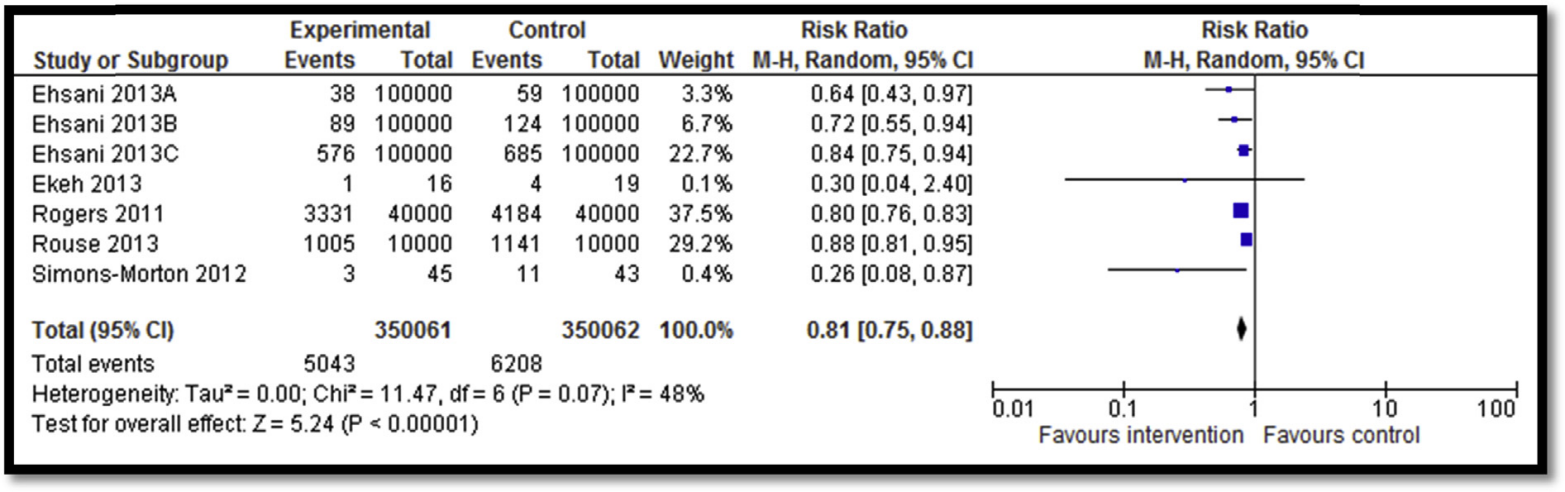
Forest plot for the impact of GDL on incidence of road accidents.

**Figure 3:**
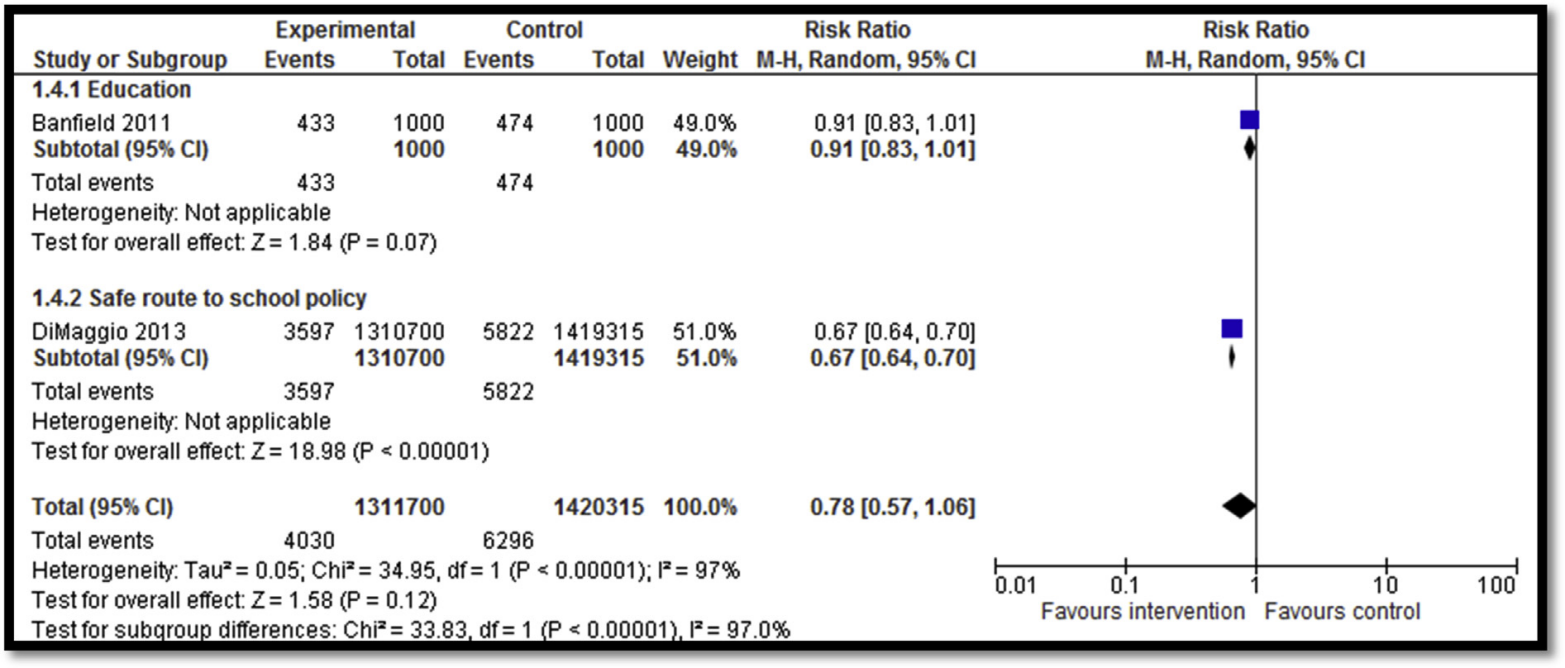
Forest plot for the impact of interventions for motor vehicle injury prevention on incidence of injuries (subgrouped according to the type of intervention).

Eleven studies [27], [28], [29], [30], [31], [35], [37], [53] focused on preventing motor vehicle injuries including graduated driver license (GDL) programs; education and awareness programs; role of effective sleep; taking safe driving routes; and guest lectures from people who had sustained debilitating injuries to educate adolescents about the life-changing impact of such injuries. Five studies reported the impact of GDL on road accidents suggesting a significant decrease by 19% (RR: .81; 95% confidence interval [CI]: .75–.88; n = 5; Figure 2). GDL included two licensing levels of restrictions on teens' driving before they are eligible to drive without restrictions. The first level is a learner license that allows teens to gain driving experience under the supervision of a fully licensed driver (i.e., a parent or parent-designated adult). The second level is an intermediate license that allows teens who have gained experience driving with a learner license to drive independently but with restrictions that limit their exposure to the highest risk driving conditions (i.e., at night and with young passengers). Outcome quality was rated to be low due to study design limitations since only two studies were RCTs while three were before–after studies. Four of five studies included in the meta-analysis suggested benefit. There was moderate heterogeneity. Incidence of injuries was reported by two studies focusing on Safe Route to School (SRTS) Program and hospital-based education. Overall, there was no statistically significant impact on incidence of road injuries (RR: .78; 95% CI: .57–1.06; n = 2; Figure 3). Subgroup analysis according to the type of intervention suggests that SRTS program to build sidewalks, bicycle lanes, safe crossings, and improve signage had a significant impact on reducing incidence of injuries while hospital-based one-day injury prevention education program for students did not have any significant impact on the incidence of injuries. Three studies reported helmet use after school-based training and education pertaining to bicycle safety, motor vehicle safety, and impact of injuries on lifestyle and family life and showed nonsignificant impact (RR: 1.0; 95% CI: .98–1.02; n = 3). Outcome quality was rated as “low” due to limitations in study design since all three studies were before–after studies while details of follow-up were not clear in one study. There was inconsistency in the meta-analysis since only one study suggested benefit. Three studies reported seat belt use after school-based training and education pertaining to bicycle safety, motor vehicle safety, and impact of injuries on lifestyle and family life, showing nonsignificant impact on use (RR: .99; 95% CI: .97–1.0; n = 3). Outcome quality was rated to be “low” due to study design limitation since all three studies were before–after studies and highly heterogeneous and none showed benefit.

### Interventions focusing on sports-related injury prevention

**Figure 4:**
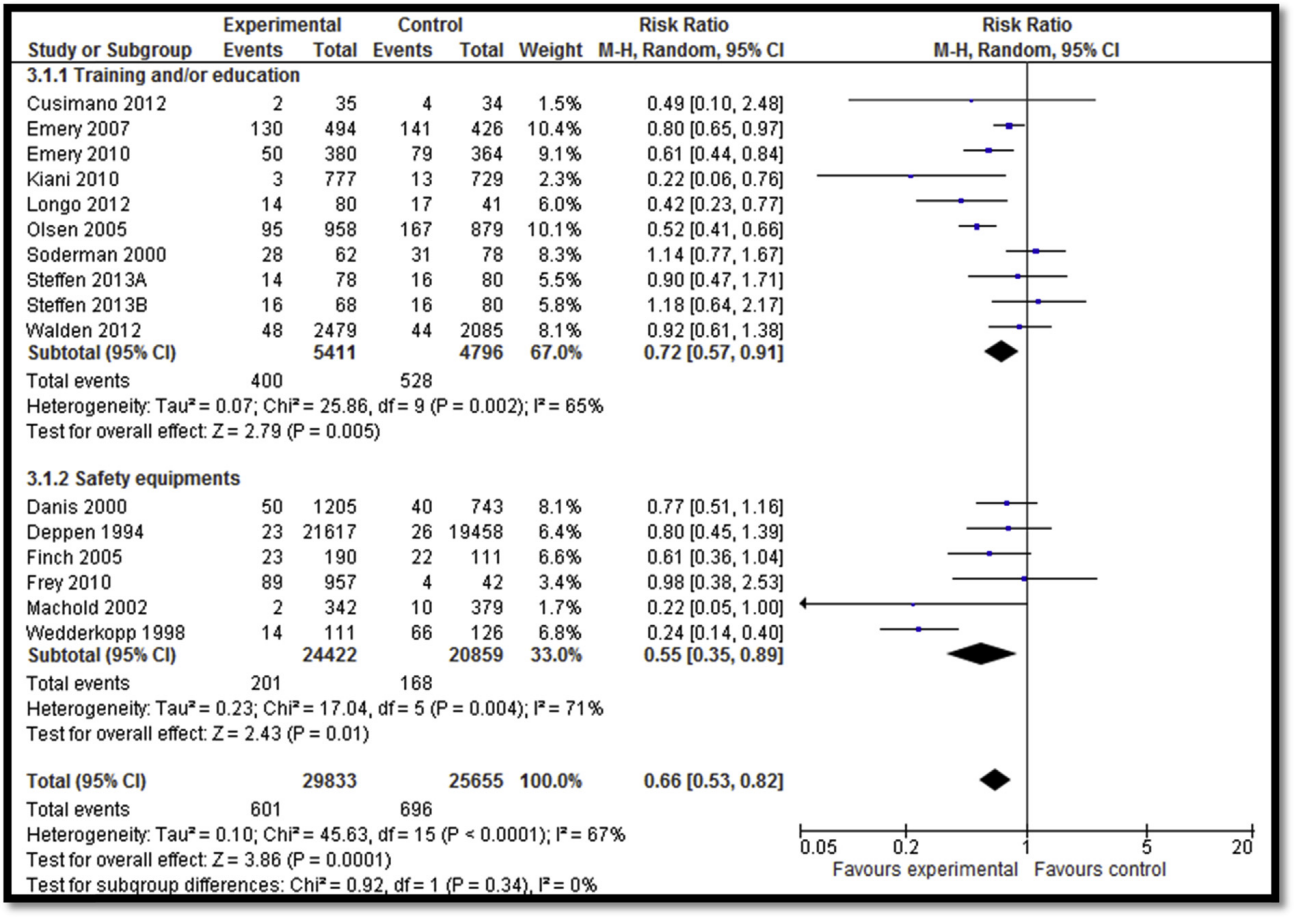
Impact of sports-related injury prevention interventions on incidence of injuries (subgrouped according to the type of intervention).

**Figure 5:**
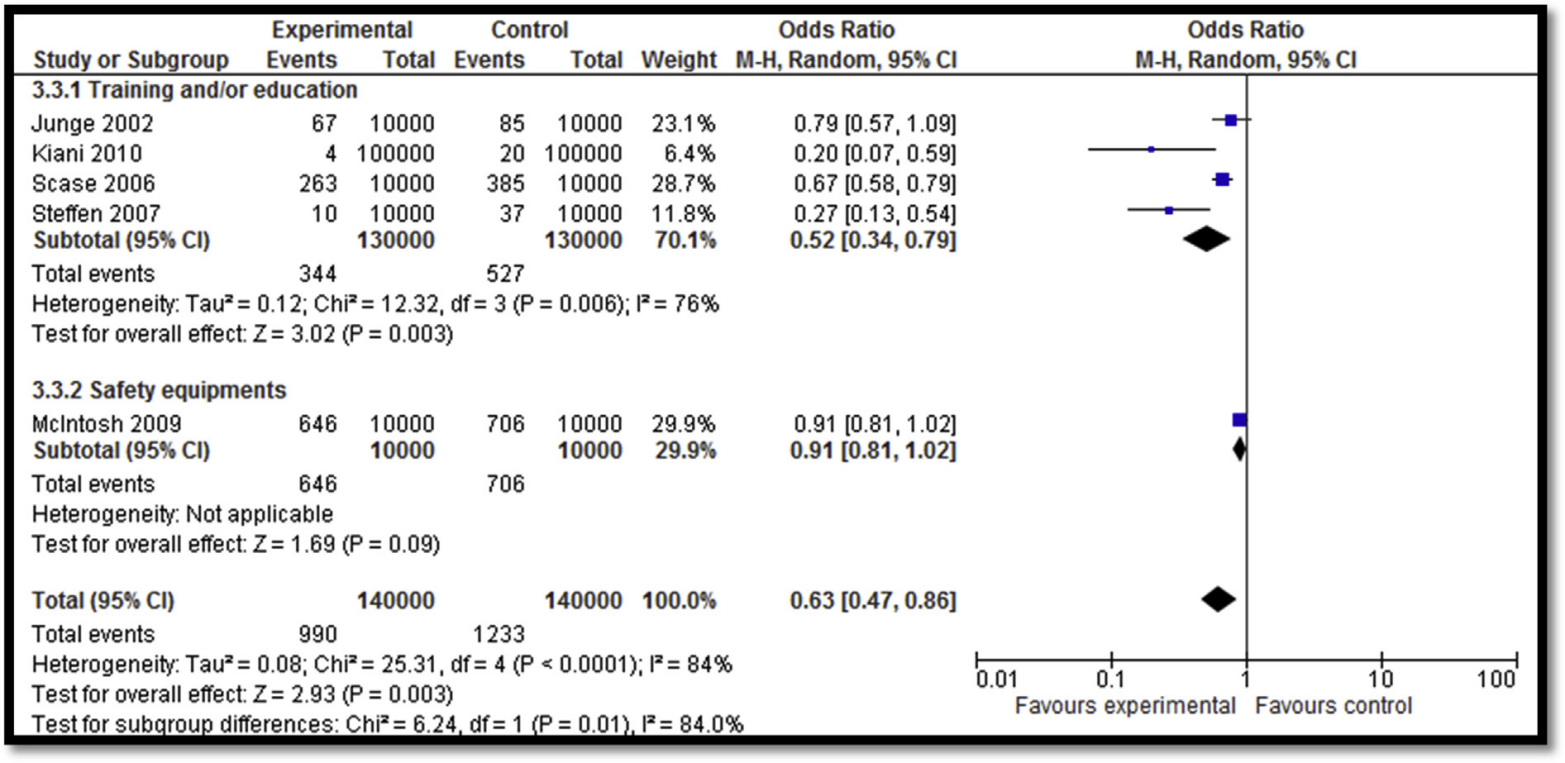
Impact of sports-related injury prevention interventions on incidence of injuries per hour of exposure (subgrouped according to the type of intervention).

**Figure 6:**
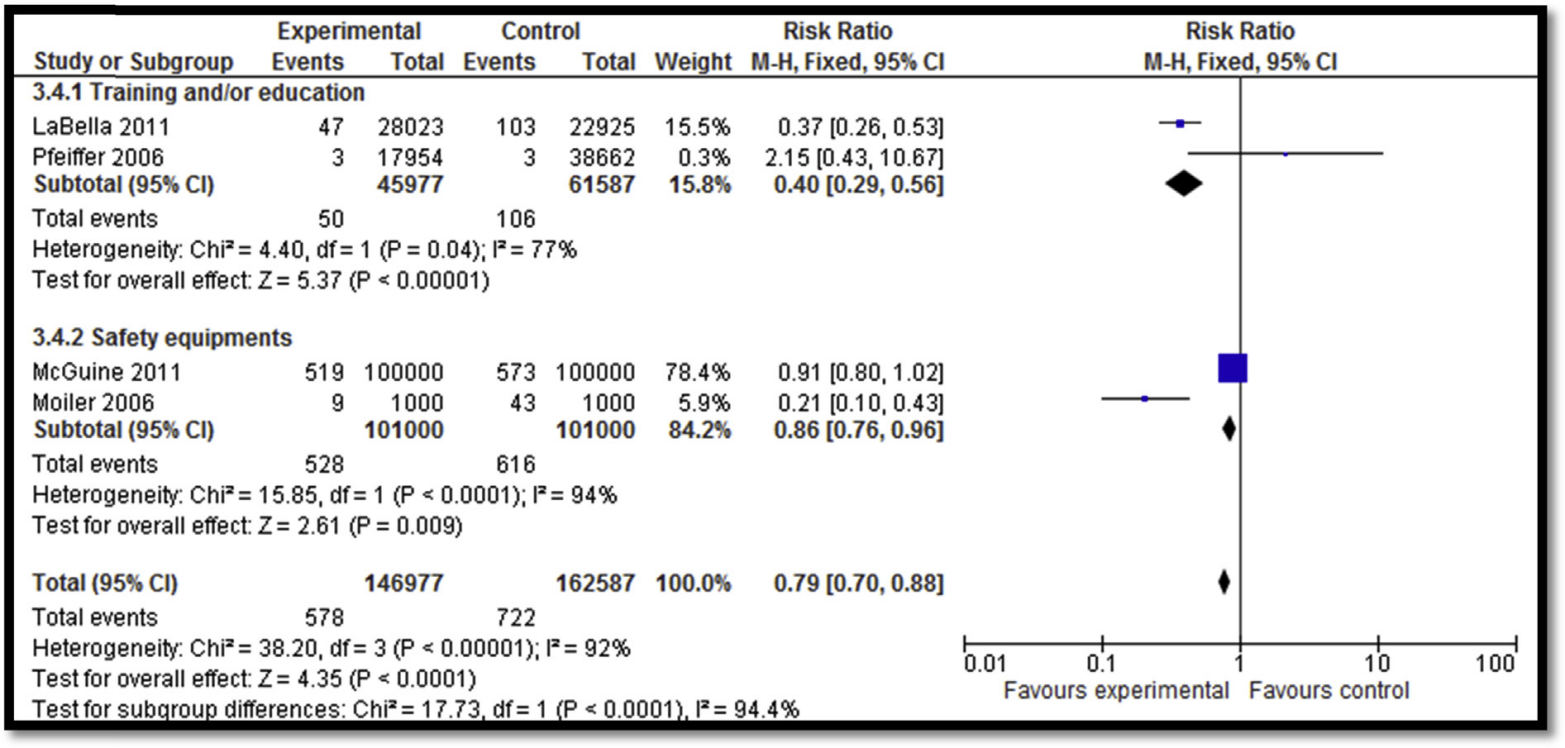
Impact of sports-related injury prevention interventions on incidence of injuries per number of exposure (subgrouped according to the type of intervention).

Twenty-four [32], [33], [36], [38], [39], [40], [41], [42], [43], [44], [45], [47], [48], [49], [50], [51], [52], [54], [55], [56], [57], [58], [59] of the included studies focused on sports-related injury prevention interventions including education and awareness sessions, training session, exercises, warm-up sessions, and use of safety equipment. Overall, sports-related injury prevention interventions lead to a decreased incidence of injuries (RR: .66; 95% CI: .53–.82; n = 15) while subgroup analysis according to the type of interventions suggests that both training ± education and use of safety equipment led to significant reductions in the incidence of injuries (Figure 4). Outcome quality was rated to be “moderate” due to study design limitation since four studies lacked adequate randomization while six studies did not have adequate blinding. There was inconsistency in the meta-analysis since 6 of 15 studies reviewed suggested benefit. There was a significant decrease in the overall incidence of injuries per hour of exposure (RR: .63; 95% CI: .47–.86; n = 5); however, the subgroup analysis suggests that the decrease was significant for training ± education and nonsignificant for the equipment use (e.g., head gear) subgroup (Figure 5). Outcome quality was rated to be “low” due to study design limitations since three of the studies did not have adequate randomization while four studies were not adequately blinded. There was considerably high heterogeneity and inconsistency since three of the five studies suggested benefit. Sports-related injury prevention led to an overall decline in injuries per number of exposures (RR: .79; 95% CI: .70–.88; n = 4) with significant impacts noted for both the training ± education and equipment use subgroups (Figure 6). Outcome quality was rated to be “low” due to study design limitations since three studies did not have adequate randomization while four studies were not adequately blinded. Three of the four studies suggested benefit; however, there was substantial heterogeneity.

## Discussion

Our review suggests that among interventions for motor vehicle injuries, GDL programs are effective in preventing road accidents. We did not find any impact of SRTS program and hospital-based training programs on the incidence of injuries. There was no impact of school-based training and education on seat belt use and helmet use. Sports-related injury prevention interventions have significant impact on reducing the incidence of injuries, injuries per hour of exposure, and injuries per number of exposures. Subgroup analysis according to the type of intervention suggests that training ± education and use of safety equipment are effective in reducing injuries. These interventions were delivered in either school or community settings underscoring the effectiveness of these delivery platforms for targeting high-risk groups. We did not find any study that evaluated interventions to prevent suffocation, drowning, poisoning, burns, or falls among the adolescent age group.

Some limitations should be recognized in our review. Since all the included studies in this review were conducted in HICs (with one exception), the review is limited by lack of data from LMICs. Although this significantly limits the generalizability of these findings, the interventions identified could be replicated in an LMIC context to evaluate effectiveness and scale-up. Included studies reported different units of exposures for the outcomes, and hence some interventions could not be pooled for analysis. There is a need to standardize the outcomes for injury prevention studies to enable comparisons of the available options. Furthermore, our review focused on interventions directed toward adolescents (i.e., 11–19 years) only; other interventions directed toward caregivers and other populations have been evaluated, and some shown to be effective in reducing child injury [9]. These should also be considered in the evidence mix for implementation.

Although awareness of injury as a major contributor to morbidity and mortality on a global scale has recently gained momentum with the World Report on Child Injury Prevention [8], injury prevention programs are limited in LMIC settings. There needs to be a movement to integrate appropriate programs into mainstream child and adolescent health initiatives. Failure to invest in programs for preventing unintentional injuries in adolescents will further increase the number of dependents in coming generations and negatively influence the health of future generations. It is imperative to involve policy makers in evaluation and implementation of optimal approaches to injury prevention. Existing evidence suggests that GDL systems, enforcement of minimum drinking age laws, wearing motorcycle and bicycle helmets, seat belt, child-restraint and helmet laws, reducing speed around schools, residential areas, and play areas are all potential interventions that should be considered for integration into policies [8], [56], [57], [58], [59]. Enforcement and better compliance with evidence-based policies could be effective and cost saving while simultaneously reducing the global burden of unintentional injuries among adolescents [60].

Unintentional injuries among adolescents continue to compromise the health of this group of children, especially in LMICs. They lead to lifelong disabilities and contribute to disability adjusted life years lost. Moreover, unintentional injuries have a greater negative economic impact in developing countries [1]. The cost of preventing unintentional injuries is much lower than the cost of treating their direct and indirect consequences. Such costs can include direct costs of medical care, hospitalization, insurance, vehicle repair, legal, school absenteeism, and lost caregiver income. Long-term economic costs should consider premature death, rehabilitation, loss of healthy years in children (permanent disabilities), and the inability of those with serious disabilities to work to the full extent [61].

Future research endeavors should focus on evaluating what works specifically in LMICs. Once implemented, there is a need for good-quality data monitoring and surveillance systems to capture the impact on the actual burden of disease and context-specific risk factors. With few LMICs having descriptive data on injuries among adolescents, there is a dire need to include “injuries” as an indicator in the health information systems at both local and national levels to monitor and direct strategies targeting this vulnerable group [8]. The Centers for Disease Control and Prevention highlights the need of future research in three domains: (1) foundational research (i.e., how injuries occur); (2) evaluative research (i.e., what works and what does not work to prevent injuries); and (3) translational research (i.e., how to put proven injury prevention strategies into action) [9].

To conclude, GDL programs are effective in preventing motor vehicle injuries while sports-related injury prevention interventions have shown significant impacts on the incidence of injuries, injuries per hour of exposure, and injuries per number of exposures. The existing evidence is mostly from HICs, limiting the generalizability of these findings for LMICs. Studies evaluating these interventions need to be replicated in an LMIC context to evaluate effectiveness with standardized outcome measures.

## Figures and Tables

**Table 1:** Characteristics of included studies

Study	Study design	Country	Setting	Intervention	Target population	Control group	Outcomes assessed
Allabaugh et al. [24]	Before–after	United States	School	Injury prevention education through the Trauma Nurses Talk Tough (TNTT). The program was presented to more than 50 schools and was also made available through the injury prevention program at our institution and was free of charge to all schools. In the sixth- to eighth-grade program, the students were educated on the consequences of using alcohol and other drugs while participating in recreational activities. Both bicycle safety and motor vehicle safety comprised a large portion of the content. The program for 9th- to 12th-grade students had similar content, but more graphics were shown in the slides, and there was more emphasis on how choices could have lifelong consequences via one quick and preventable incident. The stories were a progression of photographs taken before the incident, at the scene, in the hospital, and in the rehabilitation settings.	Students Grades 6th to 10th	No control	Helmet and seat belt use
Banfield et al. [31]	Quasi trial	Canada	Hospital	One-day injury prevention education program. Students follow the course of injury from occurrence through transport, treatment, rehabilitation, and community reintegration. They interact with a team of health care professionals and members of the emergency medical system that includes a paramedic, a police officer, nurses, a physician, and a social worker. The students are given information about the following: basic anatomy and physiology; the mechanics of injury; the effect that alcohol and drugs have on decision-making; risk assessment; concentration and coordination; the nature of injuries that can be repaired and those that cannot; and the effect of injury on families, finances, and future plans.	Adolescents 15–19 years old	No intervention	Incidence of traumatic injuries
Barbic et al. [32]	RCT	Canada	School	Special mouth guard to prevent concussions. The athletic therapist, trainer, or sports medicine physician for each team was provided with an injury report binder to document observed concussions and dental trauma. Prior to the start of the trial, these professionals were trained by the investigators in the steps necessary for concussion diagnosis and data recording.	University athletes aged 16–22 years	Normal mouth guard	Incidence of concussions
Cusimano et al. [33]	RCT	Canada	School	The intervention consisted of a 20-minute video entitled A Little Respect: ThinkFirst! It focused on the Alpine Responsibility Code, proper helmet use and clothing attire, trail and terrain sign interpretation, and emergency procedures in the event of an injury. Students also received an information brochure containing safety information about skiing and snowboarding.	Grade 7 students	General injury prevention education	Incidence of snowboarding or skiing injuries
Danis et al. [34]	Quasi trial	United States	School	Mandatory faceguards in addition to helmets during baseball	Youth baseball league players	Voluntary use of faceguards	Incidence of oculofacial injuries
Davis et al. [35]	Quasi trial	United States	Community	Half were scheduled to return for a morning appointment in about a week after obtaining a full (8.5-hour) night's sleep the evening before. That visit would be followed by a morning appointment about 2 weeks after the initial visit following a restricted (4-hour) night's sleep the evening before. The other half of the sample had the order reversed, with sleep restriction scheduled first and a full night's sleep second.	Adolescents 14–15 years old	Acute sleep deprivation	Virtual reality accidents
Deppen and Landfried [36]	Quasi trial	United States	Community	Prophylactic knee braces for football players	Male high-school football players 16–18 years old	No knee braces	Number of injuries
DiMaggio and Li [25]	Before–after	United States	Community	Safe Route to School (SRTS) program to build sidewalks, bicycle lanes, and safe crossings, improve signage, and make other improvements to built environment to allow children to more safely travel to school	School children 5–19 years old	No intervention	Number of injuries
Ehsani et al. [26]	Before–after	United States	Community	Graduated driver licensing programs that restrict driving permissions for amateur drivers	Adolescents drivers aged 16–18 years	No control	Incidence of car crashes
Ekeh et al. [37]	RCT	United States	School	Graduated Driver Licensing Program to restrict permissions for amateur drivers	High-school students who had recently received their driving license	No intervention	Incidence of car crashes
Emery et al. [39]	RCT	Canada	School	Extended warm-up with additional wobble board training	Basketball players 12–18 years old with no recent injuries	Basic training	Incidence of injuries
Emery and Meeuwisse [38]	RCT	Canada	School	The training programme was a soccer-specific neuromuscular training programme including dynamic stretching, eccentric strength, agility, jumping, and balance (including a home-based balance training programme using a wobble board) to reduce basketball injuries	Soccer players 13–18 years old with no recent injuries	Basic aerobic training	Incidence of injuries
Falavigna et al. [27]	Before–after	Brazil	School	The intervention was presented in audiovisual form and was divided into two periods; initially, a video was shown with an unintentionally injured young victim, who reported the experience of being injured and the impact on his lifestyle and his family life; then, a brain and spinal cord trauma prevention lecture was given based on the Pense Bem Project. General guidelines were given about attitudes toward prevention of neurotrauma (never drink and drive [take a taxi or bus, or call your parents to pick you up]; and follow this rule: everyone must wear a seat belt in your car). The lecture time was approximately 60 minutes.	High-school students	No intervention	Helmet and seat belt use
Finch et al. [40]	RCT	Australia	Community	Custom-made mouth guards for each athlete	Male football players aged 16–28 years	Usual mouth guards	Incidence of injuries
Frey et al. [41]	RCT	United States	School	Ankle braces to prevent injuries	High-school volleyball players	No braces	Incidence of ankle injuries
Junge et al. [42]	Quasi trial	Switzerland	Community	Exercise and education program for players and coaches	Male soccer players aged 14–19 years old	No intervention	Incidence of injuries per 1,000 hours
Kiani et al [43]	Quasi trial	Sweden	Community	Injury risk awareness, structured warm-up, and strengthening exercises	Female soccer players aged 13–19 years old	No intervention	Incidence of knee injuries
Koestner [28]	Before–after	United States	School	Educational seminar in three phases. On Day 1, the students watched a 15-minute video, “Think About Your Choices,” which features honest and direct testimonies from individuals who have sustained serious brain or spinal cord injuries. Phase 2 included a brief discussion led by a trauma nurse, using the TFFT PowerPoint presentation on anatomy of the brain and spinal cord along with information on the mechanism of injury and strategies to prevent injuries.	High-school students aged 14–15 years	No control	Incidence of helmet and seat belt use
LaBella et al. [44]	RCT	United States	School	Structured neuromuscular warm-up	Females high-school soccer and basketball players	No intervention	Incidence of injuries
Longo et al. [45]	RCT	Not clear	Community	Injury prevention training and warm-up program	Male basketball players aged 11–19 years old	No intervention	Incidence of injuries
Machold et al. [46]	RCT	Austria	School	Biomechanically constructed wrist protectors	High-school students going skiing or snowboarding	No intervention	Incidence of severe wrist injuries
McGuine et al. [47]	RCT	United States	School	Ankle braces fitted to each player	Male football players Grades 9–12	No intervention	Incidence of injuries
McIntosh et al. [48]	RCT	Australia	Community	Mandatory padded head gear	Male rugby players aged 12–21	No compulsory head gear	Incidence of injuries and concussions per 1,000 hours
Moiler et al. [49]	Quasi trial	Australia	Community	Fibular repositioning tape applied by research assistants using a standardized method	Male basketball players aged 13–23	No intervention	Incidence of ankle injuries per 1,000 exposures
Olsen et al. [50]	RCT	Norway	Community	Structured warm-up, training, and fitness education program	Handball players aged 15–17 years old	No intervention	Incidence of knee and ankle injuries
Pfeiffer et al. [51]	Quasi trial	United States	School	Structured warm-up and training programs	Females high-school athletes	No intervention	Incidence of injuries
Rogers et al. [29]	Before–after	United States	Community	Graduated Driver Licensing Program to restrict permissions for amateur drivers	Adolescents drivers	No control	Incidence of car crashes
Rouse et al. [30]	Before–after	United States	School	Graduated Driver Licensing Program to restrict permissions for amateur drivers	Drivers under the age of 19 years	No control	Incidence of car crashes
Scase et al. [52]	Quasi trial	Australia	Community	Landing, falling, and recovery skills training	Australian male football players <18 years old	No intervention	Incidence of injuries per 1,000 hours of exposure
Simons-Morton and Winston [53]	RCT	United States	Community	Reducing the exposure of novice teen drivers to high-risk driving conditions-graduated driver licensing policy and parental management of novice teen drivers	Newly licensed drivers <18 years old	G-force measurements without detailed feedback	Incidence of car crashes and high-risk events
Soderman et al. [54]	RCT	Sweden	Community	Balance board training	Female soccer players aged 15–25 years old	No intervention	Incidence of injuries
Steffen et al. [56]	RCT	Norway	Community	Structured training exercises to improve stability and balance	Female soccer players aged 13–17 years old	Routine warm-up	Incidence of injuries
Steffen et al. [55]	RCT	Canada	Community	Structured warm-up and training for athletes and an educational workshop for coaches	Female football players aged 13–18 years old	Injury prevention training program without physiotherapist or basic guidance about injury program to coach without actual implementation	Incidence of injuries
Walden et al. [57]	RCT	Sweden	Community	Structured neuromuscular warm-up and stability exercises	Female handball players aged 12–17 years old	No intervention	Incidence of knee injuries
Wedderkopp et al. [58]	RCT	Not clear	Community	Structured warm-up and training using ankle disks	Female handball players aged 16–18 years old	No intervention	Incidence of injuries

RCT = randomized controlled trial.

**Table 2:** Summary of findings for the effect of interventions for motor vehicle injury

Quality assessment						Summary of findings		
Number of studies	Design	Limitations	Consistency	Directness		Number of participants		SMD/RR (95% CI)
				Generalizability to population of interest	Generalizability to intervention of interest	Intervention	Control	
Helmet use: low outcome-specific quality of evidence								
Three	Before–after	Reliability not clear in two studies, details of follow-up not clear in one study.	Only one study suggests benefit No heterogeneity, *I*^2^ = 0%	All studies aimed at improving safety in adolescents	Interventions to increase awareness	1,174	1,162	1.00 (.98–1.02)
Seatbelt use: low outcome-specific quality of evidence								
Three	Before–after	Reliability not clear in two studies, details of follow-up not clear in one study.	No study suggests benefit Considerable heterogeneity, *I*^2^ = 78%	All studies aimed at improving safety in adolescents	Interventions to increase awareness	1,622	1,588	.99 (.97–1.00)
Incidence of road accidents: low outcome-specific quality of evidence								
Five	RCT and before–after	Only two studies were randomized	Four studies suggest benefit Moderate heterogeneity, *I*^2^ = 48%	All studies aimed at improving safety in adolescents	Interventions to increase safe driving for all adolescents	5,043	6,208	.81 (.75–.88)

CI = confidence interval; RCT = randomized controlled trial; RR = relative risk; SMD = standard mean difference.

**Table 3:** Summary of findings for the effect of interventions focusing on sports-related injury prevention

Quality assessment						Summary of findings		
Number of studies	Design	Limitations	Consistency	Directness		Number of participants		SMD/RR (95% CI)
				Generalizability to population of interest	Generalizability to intervention of interest	Intervention	Control	
Incidence of injuries: moderate outcome-specific quality of evidence								
15	RCT and before–after studies	Four studies not randomized, six studies not adequately blinded	Six studies suggest benefit Considerable heterogeneity, *I*^2^ = 75%	All studies aimed at improving safety in adolescents	Interventions to prevent injuries included increasing awareness and performing preventive exercises	1,034	1,170	.66 (.53–.82)
Incidence of injuries per hours of exposure: low outcome-specific quality of evidence								
5	RCT and before–after studies	Three studies not randomized, four studies not adequately blinded	Three studies suggest benefit Substantial heterogeneity, *I*^2^ = 84%	All studies aimed at improving safety in adolescents	Interventions to prevent injuries included increasing awareness and performing preventive exercises	990	1,233	.63 (.47–.86)
Incidence of injuries per number of exposures: low outcome-specific quality of evidence								
4	RCT and before–after studies	Three studies not randomized, four studies not adequately blinded	Three studies suggest benefit Substantial heterogeneity, *I*^2^ = 92%	All studies aimed at improving safety in adolescents	Interventions to prevent injuries included increasing awareness and performing preventive exercises	4,175	6,544	.79 (.70–.88)

CI = confidence interval; RCT = randomized controlled trial; RR = relative risk; SMD = standard mean difference.

## Box 1 Levels of quality of a body of evidence in the GRADE approach

**Table:**

Underlying methodology	Quality rating
Randomized trials or double-upgraded observational studies	High
Downgraded randomized trials or upgraded observational studies	Moderate
Double-downgraded randomized trials or observational studies	Low
Triple-downgraded randomized trials or downgraded observational studies or case series/case reports	Very low

GRADE = Grading of Recommendations Assessment, Development and Evaluation.
